# Enigmatic Histamine Receptor H_4_ for Potential Treatment of Multiple Inflammatory, Autoimmune, and Related Diseases

**DOI:** 10.3390/life10040050

**Published:** 2020-04-24

**Authors:** Pakhuri Mehta, Przemysław Miszta, Przemysław Rzodkiewicz, Olga Michalak, Piotr Krzeczyński, Sławomir Filipek

**Affiliations:** 1 Faculty of Chemistry, Biological and Chemical Research Centre, University of Warsaw, 02-093 Warsaw, Poland or pakhurimehta@gmail.com (P.M.); pmiszta@chem.uw.edu.pl (P.M.); 2 Department of General and Experimental Pathology, Medical University of Warsaw, 02-091 Warsaw, Poland; przemyslaw.rzodkiewicz@wum.edu.pl; 3 Łukasiewicz Research Network-Pharmaceutical Research Institute, 01-793 Warsaw, Poland; o.michalak@ifarm.eu (O.M.); p.krzeczynski@ifarm.eu (P.K.)

**Keywords:** histamine H_4_ receptor, G protein-coupled receptors, allergic diseases, inflammatory diseases, autoimmune disorders, neuropathic pain, cancer

## Abstract

The histamine H_4_ receptor, belonging to the family of G-protein coupled receptors, is an increasingly attractive drug target. It plays an indispensable role in many cellular pathways, and numerous H_4_R ligands are being studied for the treatment of several inflammatory, allergic, and autoimmune disorders, including pulmonary fibrosis. Activation of H_4_R is involved in cytokine production and mediates mast cell activation and eosinophil chemotaxis. The importance of this receptor has also been shown in inflammatory models: peritonitis, respiratory tract inflammation, colitis, osteoarthritis, and rheumatoid arthritis. Recent studies suggest that H_4_R acts as a modulator in cancer, neuropathic pain, vestibular disorders, and type-2 diabetes, however, its role is still not fully understood.

## 1. Introduction

Histamine action via distinct receptors (H_1_R–H_4_R) modulates diverse physiological as well as pathological processes. Due to their differential receptor pharmacology and signal transduction properties, histamine has characteristic effects dependent upon the histamine receptor subtype it is bound to. Histamine receptors H_1_–H_4_ are widespread throughout the body but there is limited knowledge about the H_4_R. The role of H4R in neuropathic pain transmission and other diseases is still controversial after nearly 20 years since its discovery. This may be due to biased signaling of histamine and H_4_ receptor agonists and differential effects on multiple signaling pathways in central and peripheral parts of the sensory nervous system. However, in the last two decades, there was a particular increment in evidence supporting participation of H_3_R and H_4_R in neuropathic pain modulation [1]. Histamine has also been identified to be responsible for a vascular type headache, e.g., migraine, hence the antihistamines are regarded as a possible treatment [2]. The proper action of particular subtypes of histamine receptors is of special importance as it has been shown for instance for the delirium syndrome in which H_1_R and H_2_R antagonists have pro-delirium potential, while H_3_R antagonists have proved to be beneficial in combating delirium. The H_4_R may also play an indirect role requiring further intensive exploration [3].

Pulmonary fibrosis is the most frequent form of interstitial lung disease. Unavailability of effective therapies has led to the urge of exploiting novel curative approaches. Histamine receptor H_4_ has been recognized as a new target for inflammatory and immune diseases, and H_4_R ligands reduced inflammation and oxidative stress in lung tissue. It has been shown that poly(ADP-ribose) polymerase (PARP-1) and H_4_R are both involved in inflammatory and fibrotic responses. Treatment with H_4_R antagonist JNJ7777120 ((5-chloro-1H-indol-2-yl)(4-methyl-1-piperazinyl)-methanone; CAS Number 459168-41-3; Molecular Weight: 277.8) in a condition of PARP-1 inhibition, provides anti-inflammatory and anti-fibrotic effects, causing reduction in airway remodeling and bronchoconstriction. Its synergistic effect with selective PARP-1 inhibitors could be of potential use for the treatment of pulmonary fibrosis [4]. Viral infections can be important contributors to development of asthma and chronic obstructive pulmonary disease. Pulmonary fibrosis is the main factor leading to pulmonary dysfunction and quality of life decline in SARS survivors. Gaining a deeper understanding of the interaction between Coronaviruses and the innate immune system of the host may shed light on the development and persistence of inflammation in the lungs and can possibly reduce the risk of lung inflammation caused by CoVs [5].

## 2. The Histamine Receptors—Localization and Function

Histamine receptors, numbered in the order of their discovery H_1_R-H_4_R, are G protein-coupled receptors (GPCRs) that constitute the largest family of cell surface receptors in humans and play a key role in cellular signaling. In the central nervous system (CNS), the histaminergic system is mainly modulated by histamine, an inflammatory biogenic amine involved in wide range of pathophysiological effects through interaction with histamine GPCRs which belong to class A (rhodopsin-like) GPCRs. These GPCRs differ in localization and cellular signaling mechanisms and they even differ in the level of constitutive activity, i.e., the ability to adopt an active conformation independent of ligand binding [6,7]. H_1_R and H_2_R are found in the brain and periphery, H_3_R is abundant in the CNS, while H_4_R has low expression, if any, in the CNS and is predominantly expressed on a variety of peripheral immune cells such as eosinophils, dendritic cells, mast cells (HMC-1, LAD-2, and primary cord blood derived CD34+ human mast cells), leukocytes, and T-cells (including γδT, T helper 1, 2, Th17, and CD8 cells) [6,8,9,10,11,12]. The presence and role of H_4_R in brain nervous tissue is yet elusive and not fully known but the presence of H_4_R in non-neuronal cells in the brain has been confirmed [13,14]. Functional H_4_ receptors that increase [^35^S]-GTPγS binding and/or decrease noradrenaline release have not been identified in human, guinea pig, and mouse cortex [15]. In human mast cells, H_4_R mediates release of cytokines, leukotrienes, and chemokines (TGF-β1, TNF-α, TNF-β, PDGF-BB, TIMP-2, M-CSF, IP-10, IL-16, IL-6, IL-3, IL-10, MIP-1α, IL-1α, ICAM-1, Eotaxin-2, RANTES, IL-8, MCP-1, and IL-6sR) [10].

**Figure 1:**
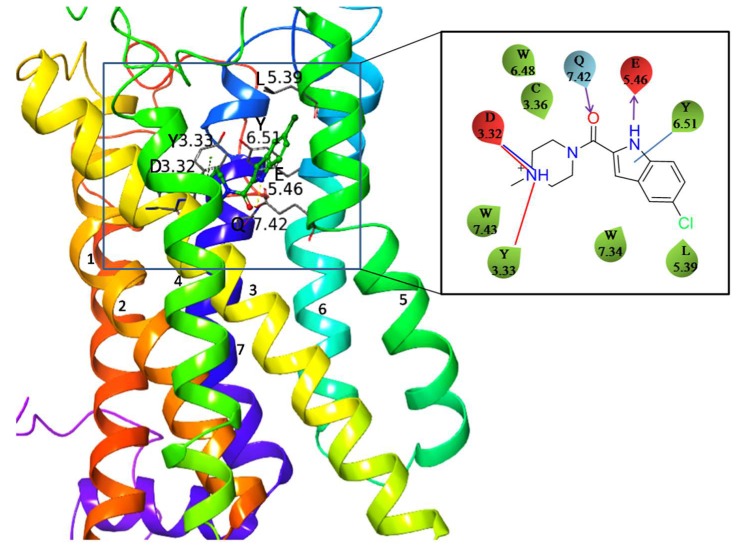
The homology model of H_4_R with docked JNJ7777120 antagonist. The specific ligand–receptor interactions are shown on the right panel. D3.32 forms both a hydrogen bond and an ionic interaction with the charged amine group of the ligand.

Being a member of the most populated class A of the GPCR superfamily, human H_4_R also contains seven transmembrane helices and a short amphipathic helix that possibly runs parallel to the cytosolic membrane surface. It consists of 390 amino acid residues possessing all of the highly conserved sequence motifs [16,17] of the class A GPCRs including the most evolutionary conserved residues in each of the transmembrane helices: N1.50, D2.50, R3.50, W4.50, P5.50, P6.50, and P7.50 (Ballesteros–Weinstein numbering [18]) indicating the same activation mechanism of H_4_R as that of the other receptors in class A GPCRs [19]. The Ballesteros–Weinstein numbering scheme of GPCRs provides information about the relative positions of amino acids present in seven transmembrane helices. Each residue of the receptor is recognized by two numbers separated by a dot; the first number (1–7) indicates the number of the transmembrane helix where the residue is located while the second number indicates its position in relation to the most conserved residue, assigned number 50, of the same helix. The prominent residues such as D3.32 and W7.40, specific for amine-activated GPCRs, are also present in the H_4_R [20]. It has been observed that the two agonists (histamine and OUP-16) exhibit complementary interactions with residues D3.32, E5.46, and T6.55, while the reference antagonist JNJ7777120 exhibits interactions with D3.32 and E5.46 only (Figure 1), implicating a differentiating role of T6.55 in ligand binding and receptor activation [21,22]. There are also striking complementarities between the H_4_R binding pocket and the structural properties of most H_4_R antagonists. They consist of a minimum of one, or preferably two, positively charged groups complementary to two negatively charged residues in the binding pocket, namely D3.32 and E5.46, and such double interaction is crucial for the interaction of high affinity ligands with H_4_R [21].

Among the histamine receptors, H_1_R and H_4_R possess 40% amino acid identity in the transmembrane region and they recognize the same endogenous ligand that is histamine. Due to such similarity the crystal structure of H_1_R has been used by many researchers for building the homology models of H_4_R. However, there are substantial differences in histamine receptor binding sites. For instance, N4.57 in H_4_R is equivalent to W4.56, L5.39 to K5.39, E5.46 to N5.46, and Q7.42 to G7.42 in H_1_R. Additionally, the mutations of residues N4.57 and E5.46 resulted in significant alteration of inhibition constants of JNJ7777120 which was the first reported H_4_R antagonist [23] and the homology model of H_4_R featured two specific hydrogen bonds and ionic interactions of JNJ7777120 to D3.32 and E5.46 [24]. H_4_R has the highest sequence homology with H_3_R as it possesses 37% amino acid identity in protein sequence and 58% identity in the transmembrane region. It is evident that a number of ligands of H_4_R also have a high affinity for H_3_R due to the identical amino acids within the binding site of both receptors, including E5.46, Y3.33, and Y6.51, involved in ligand binding [25]. These amino acids residues contribute to the similarity between the binding sites of hH_3_R and hH_4_R forcing similar conformations of ligands. This explains the number of ligands which are antagonists of both receptors. Additionally, various substituted histamine derivatives such as R-(α)-methylhistamine have significant H_4_R binding in addition to H_3_R [6]. Istyastono et al. have shown that the E5.46Q mutation impaired the binding strength of clobenpropit and its derivatives in both those receptors [26]. Moreover, the L5.39V and E5.46Q mutations resulted in a decrease of binding of the reported ligands to H_4_R. This finding emphasized the importance of the E5.46 residue which provides a crucial interaction with antagonists [27].

**Figure 2:**
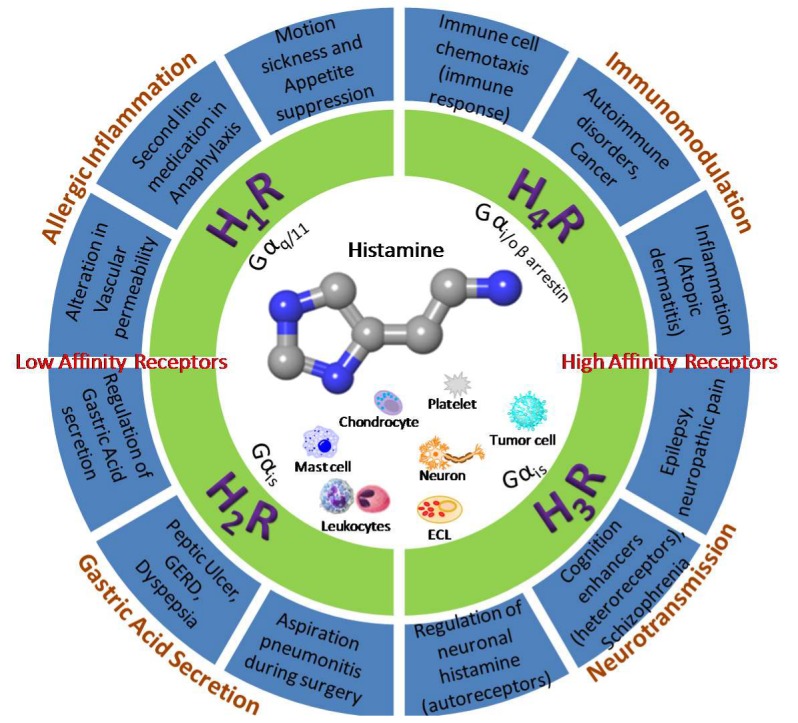
Classification of histamine receptors (H_1_R–H_4_R) in relation to their functions. H_1_R–H_3_R transduce extracellular signals via Gα_q/11_, Gα_is_, and Gα_i/o_, respectively, while H_4_R acts through Gα_i/o_ and β-arrestin. H_1_R and H_2_R are low-affinity receptors while H_3_R and H_4_R are high-affinity receptors towards histamine. Ligands of H_1_R–H_4_R have therapeutic applications in allergic inflammation, gastric acid secretion, neurotransmission, and immunomodulation, respectively. The information in the figure is partially based on [44].

A plethora of studies have related the heterogenic and complex pharmacology of histamine receptors to various diseases: H_1_R to the allergic inflammation, anaphylaxis, and motion sickness [28,29], H_2_R to the stimulation of gastric acid secretion leading to peptic ulcer, GERD and aspiration pneumonitis [30,31], H_3_R to the neurotransmission controlling sleep, cognitive processes, schizophrenia, epilepsy, and pain [32,33,34,35,36,37], and H_4_R to the immune responses (cancers, myocarditis) and inflammation [38,39,40,41,42] (Figure 2). The H_3_ and H_4_ receptors have relatively high affinity for histamine (5–10 nM) compared to the low affinity of H_1_R and H_2_R which is in the μM range [6,43]. Hence, the biological response has been linked directly with the local tissue histamine concentration and functional expression of different receptors [6].

## 3. Species Differences of H_4_R

**Scheme 1:**
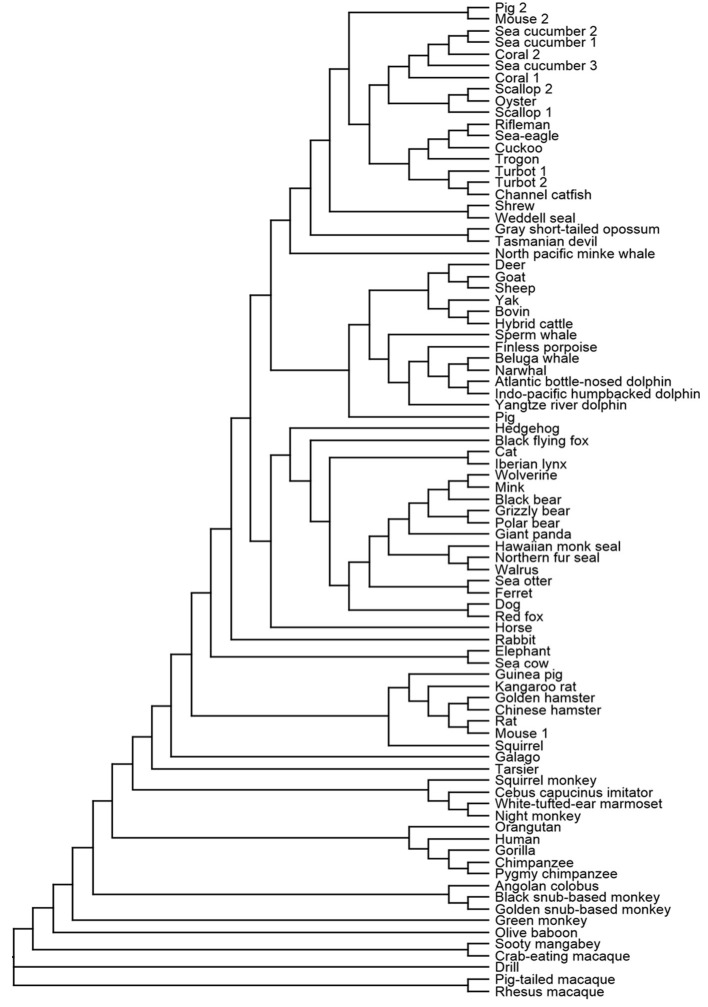
Phylogenetic tree of H_4_R orthologues. The sequences were obtained from UniProt [47] and the sequences were aligned with ClustalW and the cladogram was created with Clustal Omega service [48].

Following the identification of the human H_4_R (UniProt id: Q9H3N8), various sequences of mouse, rat, guinea pig, pig, dog, and monkey H_4_R have been reported and functionally expressed [38]. Eighty-five protein sequences of H_4_R orthologues from different species have been extracted from the UniProt database and aligned to draw the phylogenetic relationship between H_4_R orthologues (Scheme 1). The H_4_ receptors of the chimpanzee, gorilla, and orangutan show the highest sequence homology (98–99%) with the human orthologue (hH_4_R). H_4_ receptors of some species are highly homologous to hH_4_R with sequence homology between 78% and 94%, specifically those of macaques, baboon, drill, *Angolan colobus*, mangabey, *Cebus capucinus* imitator, marmoset, and *Philippine tarsier* (Table 1). Orthologues in some species were only moderately homologous to hH_4_R with sequence homology between 54% and 73% while the least homologous showed homology ranging from 10% to 47%. Pig, mouse, smooth cauliflower coral, Japanese scallop, turbot, and pig have each two H_4_R orthologues while sea cucumber has three orthologues. However, these orthologues, show only 10–36% homology to hH_4_R while all others show a substantially higher homology (>50%). As some of the sequences are still incomplete, changes in the phylogenetic tree are to be expected. Within these GPCR sequences, the typical aminergic GPCR features (D3.32 in TM3 and E5.46 in TM5) can often be found. Detailed analysis of most of these species variants is however lacking even though it could provide useful tools to dissect receptor–ligand binding. Using site-directed mutagenesis Wifling et al. have proved that the F169, located in the second extracellular loop ECL2, is a crucial amino acid for differential interactions, affinities, and potencies of certain agonists with the human and mouse H_4_R orthologues [45]. Receptor sequence differences have implications even for ligand function as the JNJ7777120 ligand acts as a partial inverse agonist at the human H_4_R, but as a partial agonist at the rat and mouse H_4_R which possess lower constitutive activity than their human counterpart. Therefore, differences in pharmacological activities of H_4_R ligands between different species might hamper preclinical development of future H_4_R drugs [46].

## 4. The Pharmacological Effects of H_4_R Ligands

**Figure 3:**
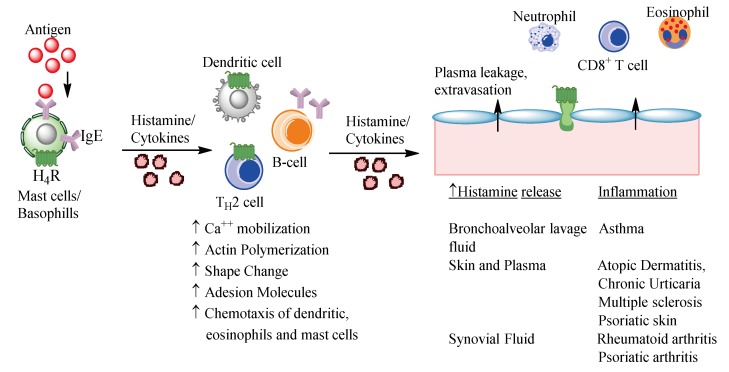
Potential role of histamine and histamine H_4_R-induced recruitment of eosinophils and mast cells in chronic allergic inflammation. Histamine has been known to be a major mediator of inflammation. Histamine H_4_ receptors are expressed on the surface of both eosinophils and mast cells. Allergen may crosslink immunoglobulin E (IgE) on mast cells to release histamine, lipid mediators, and cytokines. Antigen is also processed by dendritic cells and macrophages for presentation to T-helper cells. During this process a local release of histamine and cytokines may occur. Histamine can act on a variety of cells and at different levels. In asthma histamine can facilitate the recruitment of inflammatory cells by regulating the chemotaxis of additional dendritic cells, eosinophils, and mast cells to the airways via the action at H_4_R. Histamine may additionally affect cytokine release from CD8^+^ cells via binding to H_4_R and from eosinophils, neutrophils, and mast cells through multiple histamine receptors.

Although the pharmacology of H_4_R ligands is yet not fully elucidated H_4_R has been widely studied and reviewed since its characterization and cloning in 2000 [25,49]. The vast body of accumulating knowledge on physiological and pathophysiological functions associated with H_4_R modulation can be exploited for therapeutic purposes [11]. The properties of H_4_R make this amine receptor and its ligands of interest to specialists in the field of allergology, neurobiology, gastroenterology, endocrinology, and also to researchers of cardiovascular functions [6,50]. The results of research on the role of H_4_R in various pathophysiological and immunological processes indicate its association with the development and course of many diseases including a crucial role of H_4_R in airway and dermal inflammation (Figure 3), pruritus, ocular inflammation, arthritis, systemic lupus erythematosus, Sjogren’s syndrome, multiple sclerosis, gastric ulcer, cancer, and pain [12,51].

### 4.1. Allergic Diseases

Inflammatory conditions were for a long time thought to be mediated by activation of the histamine receptor subtype 1. However, the discovery and pharmacological characterization of H_4_R ligands especially antagonists, (and, to a lesser extent H_3_R and even H_2_R ligands) on mast cells, eosinophils, and T cells demonstrates the possibility of its involvement in inflammatory conditions/symptoms such as atopic dermatitis (AD), asthma, allergic rhinitis, rheumatoid arthritis (RA), and pruritus in humans. This is evident from the results obtained in diverse experimental models of inflammation including hepatic ischemia-reperfusion, colitis, atopic dermatitis, in which H_4_R antagonists (JNJ7777120, JNJ10191584, thioperamide) proved to be efficient anti-inflammatory agents with reduced neutrophil recruitment and release of cytokines [51,52]. Preclinical and clinical data strongly suggest the regulatory involvement of H_4_R in the calcium influx and cellular chemotaxis [53,54], hence establishing a link between the potential therapeutic application of selectively acting H_4_R ligands to inflammatory conditions while also indicating involvement of H_4_R in diseases accompanied by itch and pain [55]. The investigations of histamine in the inflammation process have led to a development of the first highly potent and selective non-imidazole H_4_R antagonist JNJ7777120, followed by reexamination and synthesis of a plethora of H_4_R-targeted compounds [50,51].

Currently, many H_4_R ligands are known, synthesized, and evaluated [56,57]. Studies using selective H_4_R ligands in animal models of pruritus revealed a role for H_4_R in mediating chronic pruritus associated with conditions such as atopic dermatitis [51,58]. Antagonists of H_4_R (JNJ7777120, JNJ39758979, INCB38579, and others) reduced pruritus in a number of animal studies [59] as well as itching sensation in different conditions in human patients. Alcaftadine, a topical ophthalmic drug indicated for the prevention of itching associated with allergic conjunctivitis, is a potent H_1_R and H_2_R antagonist (in fact, inverse agonist) with weak inverse agonistic activity also towards H_4_R [60]. Administration of H_1_R/H_4_R antagonists or co-administration of H_1_R and H_4_R antagonists will probably be effective also in humans. Such antagonists are more efficacious as compared to olopatadine (H_1_R antagonist without H_4_R activity) [61]. Consequently, these studies indicate that H_4_R is involved in mediating pruritic responses in humans, and that H_4_R antagonists are ought to be effective in the treatment of pruritic histamine-mediated conditions, such as AD, acute urticaria, allergic rhinitis, or allergic conjunctivitis.

The histamine receptor H_4_R was also found on cartilage cells–chondrocytes [62,63]. As the presence of the histamine triggering protein (HRF) has been identified in the joints of people with RA, it seems very likely that H_4_R antagonists will be used in the future in the treatment of RA [64]. This receptor may also be important in the pathogenesis of Sjörgen’s syndrome, erythematous lupus erythematosus, and atopic dermatitis [65]. H_4_R activation not only results in phosphorylation of ERK and PI3K in a time dependent manner but it also leads to enhanced synthesis of inflammatory mediators associated with allergic reactions. It leads to inflammatory conditions as well as contributes to postinflammatory visceral hypersensitivity, thus, making H_4_R antagonists important for reducing inflammation and normalizing postinflammatory visceral hypersensitivity [66].

### 4.2. Asthma

H_4_R seems to be an interesting pharmacological target in the treatment of asthma [6]. Asthma is a condition typically characterized by involvement of eosinophils and mast cells [67,68,69]. Extensive studies have provided evidence detailing the functional profile of H_4_R in eosinophil biology [70] and in the chemotaxis and differentiation of other immune cell types. In experiments carried out on animal models of inflammation of the airways, it was observed that in mice lacking the H_4_R gene, there was a significant reduction in the allergic reaction caused by the administration of a chicken protein-ovalbumin [71]. Chemotaxis of eosinophils was shown to be blocked by H_4_R selective antagonists (JNJ7777120, JNJ39758979, or JNJ10191584) in animal asthma models due to priming and T cell activation [51,72] while induced by histamine and selective H_4_R agonists (e.g., 4-methylhistamine) [72]. Some selective H_4_R antagonists in animal models of asthma proved beneficial by mediating lung function and inflammation [51,73]. In asthma animal models, H_4_R antagonists act either directly by reducing the number of T cells at the site of inflammation [74] or indirectly when it is involved in dendritic cell function driving the response [51]. However, none of the H_4_R antagonists have been introduced to treat the above disorders.

### 4.3. Diabetes

The histamine receptor H_4_ may also be a therapeutic target in diseases not directly related to inflammation. For instance, H_4_R is suggested to be important in the pathogenesis of diabetes. In streptozotocin-induced diabetic rats H_4_R is overexpressed in tubular epithelial cells [75], and administration of a H_4_R antagonist resulted in a decreased blood sugar [76]. H_4_R participates in diabetic nephropathy progression through both a direct effect on tubular reabsorption and an indirect action on renal tissue architecture via inflammatory cell recruitment. Therefore, H_4_R antagonism emerges as a possible new multi-mechanism therapeutic approach to counteract development of diabetic nephropathy [77].

### 4.4. Parkinson’s and Alzheimer’s Diseases

Evidence about the H_4_R antagonist JNJ7777120 inhibiting propagation of microglial inflammation by attenuating the release of M1 microglial cells and largely preventing the pathological progression of Parkinson’s disease-like pathology and motor dysfunction has been provided by the latest research [78]. These findings support H_4_R as a promising novel therapeutic target for Parkinson’s disease. For Alzheimer’s disease the precise mechanism of histamine-induced Alzheimer’s pathology is not well known although the increased levels of histamine in plasma and in some areas of the brain are seen in Alzheimer’s dementia brain [79]. It is known that H_3_R can regulate cognitive and memory functions in the hippocampus so it could be involved in Alzheimer’s pathology [80]. Since H_4_R is also present in the brain and its stimulation regulates neuronal functions, then stimulating H_4_ receptors may have some beneficial effects in the brain of Alzheimer’s disease patients. Recently, it has been found that clobenpropit, a selective H_3_R antagonist with partial H_4_R agonist property, caused a significant reduction in amyloid-β deposits in a rat model of Alzheimer-like brain pathology. This effect was accompanied by marked reduction in neuronal or glial reactions so such dual-action compounds may have neuroprotective properties [81].

High similarity between H_3_R and H_4_R entails considerable similarity in ligand affinities and facilitates simultaneous activation of both receptors. Dual-acting H_3_R/H_4_R ligands may exhibit therapeutic potential in diverse pathological conditions, such as neuropathic pain, cancer, Parkinson’s, and inflammatory diseases [7,82]. Dual H_3_R/H_4_R imidazole containing ligands used so far includes compounds such as imetit, immepip, clobenpropit, and thioperamide [7].

### 4.5. Autoimmune Diseases

The characterization of a histamine receptor H_4_R with putative immunomodulating properties encouraged new hopes for the translational exploitation of this new therapeutic target for the still unmet medical needs, specifically asthma, autoimmune diseases, and a host defense. Rheumatoid arthritis (RA), which is a systemic autoimmune disorder, is characterized by chronic synovitis of peripheral joints, cartilage and bone destruction followed by joint disability. It was found that histamine and Th17 cytokines induced osteoclast differentiation from monocytes and JNJ7777120 decreased the osteoclastogenesis and the osteoclastogenic role of H_4_R has been evident in patients with RA [83]. Studies in the animal model of RA have shown that the H_4_R antagonist JNJ7777120 reduces the degree and severity of joint damage and reduces the number of cells producing IL-17 in the joint, thus, significantly inhibiting the inflammatory process in joints [84]. H_4_R involvement has been also confirmed in several types of cancers: melanoma [85], breast cancer [86], pancreatic cancer [87], and colorectal cancer [88]. H_4_R can regulate the aging and apoptosis of cancer cells and blocking H_4_R by antagonists inhibits tumor cell proliferation [86]. Histamine receptors play also an important role in the pathogenesis of multiple sclerosis. It turned out that H_1_R and H_2_R play a propathogenic role while H_3_R and H_4_R may reduce the risk of the disease [89].

## 5. Clinical Trials of Drug Candidates Targeting H_4_R

Recently, H_4_R research has been gaining a lot of importance and the clinical studies were initiated for the putative therapeutic exploitation in inflammatory and allergic disorders [38] such as atopic dermatitis (AD) [59,90], pruritus, asthma, rheumatoid arthritis (RA), as well as in vestibular disease (Table 2) [91]. Toreforant (JNJ38518168), the first oral H_4_R antagonist, has been explored for the treatment of RA patients with active disease despite concomitant methotrexate therapy (phase 2 trials, ClinicalTrials.gov database entry NCT01862224 and dose range finding study NCT01679951) [92,93]. Both studies were prematurely terminated in 2014 because of the lack of efficacy. The similar phase 2 clinical studies for the same compound evaluating efficacy and safety of toreforant in patients with symptomatic uncontrolled, persistent eosinophilic asthma (NCT01823016) [94], and in patients with moderate to severe plaque-type psoriasis (NCT02295865) [95] were completed in 2015 and 2016. In the former study toreforant (at the dose tested) failed to provide any therapeutic benefit [96]. Preclinical toxicity studies of another H_4_R antagonist, JNJ39758979, provided sufficient evidence of an excellent safe profile encouraging the clinical level testing [72]. JNJ39758979 was observed to mitigate RA in the collagen-induced arthritis models (CIAM) [59]. The completed phase 2 clinical trial demonstrating its safety and effectiveness in human volunteers with persistent asthma (NCT00946569) whereas several phase 1 studies stating its safety and pharmacokinetics, as well as its effect on histamine-induced itch (pruritus) (NCT01068223) in healthy male volunteers have successfully been accomplished [97,98]. Simultaneously, the two phase 2 clinical studies were initiated to find a dose range of JNJ39758979 in patients with RA despite concomitant methotrexate therapy (NCT01480388) and patients with uncontrolled asthma (NCT01493882) but they were withdrawn in 2014 and 2015, respectively, due to the same reasons [99,100]. This adverse effect was predicted to be related with reactive metabolites of JNJ39758979 and not with H_4_R antagonism. Hence, the significant reduction in the pruritus after JNJ39758979 administration can be concluded in the way that drug-induced agranulocytosis can be most likely an off-target effect and other H_4_R antagonists could be beneficial in the treatment of AD, particularly pruritus, without serious adverse effects [101]. In the similar clinical studies, another oral, potent, and selective H_4_R antagonist ZPL3893787 has completed phase 2 clinical trials determining its safety, efficacy, and tolerability on pruritus in adult subjects with moderate to severe AD (NCT02424253) [102] and in patients with plaque psoriasis (NCT02618616) [103] in 2016 but no results for both these studies were posted on ClinicalTrials.gov. Results showed that ZPL3893787 improved inflammatory skin lesions in patients with AD, confirming H_4_R antagonism as a novel therapeutic option [90]. Additionally, in two different phase 2 trials, there is an evaluation safety and efficacy of ZPL3893787 in patients with moderate to severe AD (NCT03517566) [104] and the impact of its concomitant use along with topical corticosteroids (TCS) and/or topical calcineurin inhibitors (TCI) in patients with AD (NCT03948334) [105]. The efficacy of Seliforant (SENS-111) in patients suffering from acute unilateral vestibulopathy is currently under evaluation in Phase 2 trial (NCT03110458) [106]. The above-mentioned observations indicate a wide range of potential clinical applications of H_4_R ligands.

## 6. Challenges and Perspectives

The H_4_R research triggered serious concern as to the role of histamine in the regulation of immune (patho)physiology. It has been established that JNJ7777120 acts as an antagonist in respect to G protein-dependent signaling, but it also recruits β-arrestin to the receptor in a non-G protein-dependent manner [107]. Moreover, JNJ7777120 acts as a partial inverse agonist at the human H_4_R but as a partial agonist at the rat and mouse H_4_ receptors [46], which show a lower constitutive activity than their human counterpart [45,46,108,109]. Frequently generated controversies and even in vivo misleading results in a variety of experimental models have been the repercussions of these problems [109]. The clinical development of JNJ7777120 as a prototype experimental tool was hampered due to several setbacks that surfaced over the past two decades including: localized concerns over the receptor subtypes, ligand binding and functional selectivity, constitutive and intrinsic activity and the biased signaling [6,46,50,51,95,110], its short half-life in vivo, and the hypoadrenocorticism toxicity concerns [50]. Therefore, the experimental findings on the role of H_4_R cannot be relied upon and need thorough investigation with caution.

Although GPCR biased signaling significantly complicates drug discovery attempts, it makes a great promise to design specific ligands with minor side effects [95,111]. The precise drugs have rapidly become the center of research for therapeutic exploitation in immunopharmacology as well as clinical immunology [90,112,113]. However, in addition to H_4_R, significant evidence attributes some immunomodulatory properties to H_2_R [90,110], thus, dissection of histamine functions in the immune system becomes indispensable. Although there are many problems in H_4_R research, a significant number of studies focusing on H_4_R provide an optimistic research perspective for this new drug target.

## Figures and Tables

**Table 1:** Sequence similarities of species specific H_4_R to the human orthologue.

	Species	Scientific Name	UniProt ID	Similarity to hH_4_R
1	Human	*Homo sapiens*	Q9H3N8	-
2	Chimpanzee	*Pan troglodytes*	H2QED2	99%
3	Gorilla	*Gorilla*	G3QS38	98%
4	Pygmy chimpanzee	*Pan paniscus*	A0A2R9BQY6	98%
5	Orangutan	*Pongo abelii*	H2NW27	98%
6	Crab-eating macaque	*Macaca fascicularis*	Q3V8G8	94%
7	Pig-tailed macaque	*Macaca nemestrina*	A0A2K6D1G7	94%
8	Rhesus macaque	*Macaca mulatta*	G7NKH9	94%
9	Olive baboon	*Papio anubis*	A0A096NGN9	94%
10	Drill	*Mandrillus leucophaeus*	A0A2K5YBZ5	94%
11	Angolan colobus	*Colobus angolensis palliatus*	A0A2K5HHL6	93%
12	Sooty mangabey	*Cercocebus atys*	A0A2K5LQL7	93%
13	Black snub-based monkey	*Rhinopithecus bieti*	A0A2K6MXG3	93%
14	Golden snub-based monkey	*Rhinopithecus roxellana*	A0A2K6RWF0	93%
15	Green monkey	*Chlorocebus sabaeus*	A0A0D9RYY4	90%
16	Ma’s Night monkey	*Aotus nancymaae*	A0A2K5CHI5	90%
17	Cebus capucinus imitator	*Cebus capucinus imitator*	A0A2K5RKQ4	90%
18	White-tufted-ear marmoset	*Callithrix jacchus*	F7IT43	89%
19	Squirrel monkey	*Saimiri boliviensis*	A0A2K6TG45	88%
20	Philippine tarsier	*Tarsius syrichta*	A0A1U7UM57	78%
21	Small-eared galago	*Otolemur garnettii*	H0WYC8	73%
22	Thirteen-lined ground squirrel	*Ictidomys tridecemlineatus*	I3MG71	72%
23	Dog	*Canis lupus familiaris*	J9P1C3	71%
24	Golden hamster	*Mesocricetus auratus*	A0A1U7Q7T1	71%
25	Grizzly bear	*Ursus arctos horribilis*	A0A3Q7WBT8	70%
26	Polar bear	*Ursus maritimus*	A0A384C2G0	70%
27	Pig	*Sus scrofa*	Q8WNV9 (Pig 1)	70%
28			A0A5G2QV28 (Pig 2)	10%
29	Red fox	*Vulpes vulpes*	A0A3Q7SYT7	70%
30	Black flying fox	*Pteropus alecto*	L5K5C7	69%
31	African elephant	*Loxodonta africana*	G3STF1	69%
32	Giant panda	*Ailuropoda melanoleuca*	G1M6D3	69%
33	Chinese hamster	*Cricetulus griseus*	A0A3L7I1V9	69%
34	Horse	*Equus caballus*	F6Z8L3	69%
35	Sea cow	*Trichechus manatus latirostris*	A0A2Y9E7N3	69%
36	Rabbit	*Oryctolagus cuniculus*	G1TKW6	68%
37	Iberian lynx	*Lynx pardinus*	A0A485N8M7	68%
38	Cat	*Felis catus*	M3WE71	68%
39	Pacific walrus	*Odobenus rosmarus divergens*	A0A2U3WW63	68%
40	Rat	*Rattus norvegicus*	Q91ZY1	68%
41	Kangaroo rat	*Dipodomys ordii*	A0A1S3F272	68%
42	Hawaiian monk seal	*Neomonachus schauinslandi*	A0A2Y9GRV4	68%
43	Northern fur seal	*Callorhinus ursinus*	A0A3Q7Q9W4	67%
44	Sea otter	*Enhydra lutris kenyoni*	A0A2Y9ITU9	67%
45	Hedgehog	*Erinaceus europaeus*	A0A1S3A2Y6	67%
46	European domestic ferret	*Mustela putorius furo*	M3Y4H4	67%
47	Mouse	*Mus musculus*	Q91ZY2 (Mouse 1)	67%
48			B2ZGH2 (Mouse 2)	66%
49	Goat	*Capra hircus*	A0A452DKI0	65%
50	Sheep	*Ovis aries*	W5PBL0	65%
51	Sperm whale	*Physeter macrocephalus*	A0A2Y9F727	65%
52	Hybrid cattle	Bos indicus*Bos taurus	A0A4W2DVG0	64%
53	Yak	*Bos mutus*	L8IEJ5	64%
54	Bovine	*Bos taurus*	E1BBS2	64%
55	Guinea pig	*Cavia porcellus*	Q91ZY3	63%
56	Black bear	*Ursus americanus*	A0A452QKW6	62%
57	Yangtze river dolphin	*Lipotes vexillifer*	A0A340YGS9	61%
58	American mink	*Neovison vison*	U6CNR7	61%
59	Beluga whale	*Delphinapterus leucas*	A0A2Y9PB56	59%
60	Yangtze finless porpoise	*Neophocaena asiaeorientalis*	A0A341CIF8	59%
61	European red deer	*Cervus elaphus hippelaphus*	A0A212C702	59%
62	Indo-pacific humpbacked dolphin	*Sousa chinensis*	A0A484GQ08	57%
63	Narwhal	*Monodon monoceros*	A0A4U1FGC1	56%
64	Wolverine	*Gulo gulo*	A0A3P4RYS2	55%
65	Atlantic bottle-nosed dolphin	*Tursiops truncatus*	A0A2U3V3K5	54%
66	Gray short-tailed opossum	*Monodelphis domestica*	F6QB56	47%
67	North-Pacific minke whale	*Balaenoptera acutorostrata scammoni*	A0A452C640	46%
68	Tasmanian devil	*Sarcophilus harrisii*	G3X3P1	45%
69	Weddell seal	*Leptonychotes weddellii*	A0A2U3YB28	42%
70	White-tailed sea-eagle	*Haliaeetus albicilla*	A0A091PX74	42%
71	Trogon	*Apaloderma vittatum*	A0A091NQC4	41%
72	Cuckoo	*Cuculus canorus*	A0A091G9T7	40%
73	Turbot	*Scophthalmus maximus*	A0A2U9BJT1 (Turbot 1)	36%
74			A0A2U9C3Q1 (Turbot 2)	36%
75	Channel catfish	*Ictalurus punctatus*	A0A2D0RQW6	36%
76	Chinese tree shrew	*Tupaia chinensis*	L8YD15	35%
77	Rifleman	*Acanthisitta chloris*	A0A091MN56	31%
78	Scallop	*Mizuhopecten yessoensis*	A0A210PRL2 (Scallop 1)	26%
79			A0A210PS14 (Scallop 2)	22%
80	Oyster	*Crassostrea gigas*	K1PU39	24%
81	Coral	*Stylophora pistillata*	A0A2B4RTL0 (Coral 1)	17%
82			A0A2B4RX53 (Coral 2)	14%
83	Sea cucumber	*Stichopus japonicus*	A0A2G8KHM7 (Sea cucumber 1)	15%
84			A0A2G8L2L5 (Sea cucumber 2)	13%
85			A0A2G8JXR8 (Sea cucumber 3)	20%

**Table 2:** Details of compounds which are/have been in clinical trial studies which started/ended/terminated in the 2014–2019 period.

Compound	Clinical Indications	Phase	Status	ClinicalTrials.Gov Database Entry	Ref.
JNJ38518168 (Toreforant) 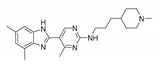	RA	2	T	NCT01862224	[92]
	RA	2	T	NCT01679951	[93]
	Asthma	2	C	NCT01823016	[94]
	Psoriasis	2	C	NCT02295865	[95]
JNJ39758979 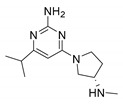	RA	2	W	NCT01480388	[99]
	Asthma	2	W	NCT01493882	[100]
ZPL3893787 (Adriforant/PF3893787/ZPL389) 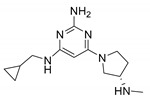	AD	2	C	NCT02424253	[102]
	Psoriasis	2	C	NCT02618616	[103]
	AD	2	R	NCT03517566	[104]
	AD	2	R	NCT03948334	[105]
SENS-111 (Seliforant) 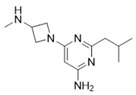	Unilateral Vestibulopathy	2	R	NCT03110458	[106]

Status: T: terminated; C: completed; R: recruiting; W: withdrawn.
